# Editorial: Evidence-based on health benefits: probiotics, micronutrients, and edible plants

**DOI:** 10.3389/fnut.2023.1272456

**Published:** 2023-10-02

**Authors:** Learn-Han Lee, Bey-Hing Goh, Piyameth Dilokthornsakul, Surasak Saokaew

**Affiliations:** ^1^Sunway Microbiome Centre, School of Medical and Life Sciences, Sunway University, Sunway City, Malaysia; ^2^Sunway Biofunctional Molecules Discovery Centre (SBMD), School of Medical and Life Sciences, Sunway University, Sunway City, Malaysia; ^3^College of Pharmaceutical Sciences, Zhejiang University, Hangzhou, China; ^4^Center for Medical and Health Technology Assessment (CM-HTA), Department of Pharmaceutical Care, Faculty of Pharmacy, Chiang Mai University, Chiang Mai, Thailand; ^5^Division of Social and Administrative Pharmacy, Department of Pharmaceutical Care, School of Pharmaceutical Sciences, University of Phayao, Phayao, Thailand; ^6^Centre of Health Outcomes Research and Therapeutic Safety (Cohorts), School of Pharmaceutical Sciences, University of Phayao, Phayao, Thailand; ^7^Unit of Excellence on Clinical Outcomes Research and Integration (UNICORN), School of Pharmaceutical Sciences, University of Phayao, Phayao, Thailand

**Keywords:** probiotics, micronutrients, edible plants, health benefits, evidence-based, SDG 3 good health and wellbeing

Nutrition is critical in preventing, treating, and prognosis acute and chronic disorders. The field of nutritional epidemiology, which has emerged from public health sciences, has been the cornerstone of nutrition research for several decades, significantly influencing dietitian practices and dietary counseling worldwide (1). Evidence-based nutrition involves a conscientious approach, working with patient's preferences and values to help them address physical, mental, and social health challenges by integrating the best available nutrition evidence with clinical expertise. In recent years, there has been growing interest in understanding the therapeutic role of probiotics, micronutrients and bioactive compounds derived from edible plants in various health conditions (2–4).

This Research Topic aims to consolidate the literature on the efficacy of probiotics, micronutrients, and edible plants in managing acute and chronic diseases. A total of 18 studies have been published on this Research Topic. Primarily, these studies contribute to the growing body of research exploring the potential benefits of nutrition-based interventions and their impact on human health. Notably, numerous research investigated the associations between dietary components and specific health outcomes, shedding light on potential interventions and preventive measures. The overarching area of interest for these 18 studies is the exploration of how dietary components influence various health conditions. Researchers have focused on examining micronutrients like zinc, copper, selenium, and manganese, as well as vitamins and antioxidants. Additionally, studies have looked at the effects of probiotics, prebiotics, and synbiotics on liver enzymes, neurological diseases, and metabolic disorders. The use of natural products derived from edible plants to ameliorate health conditions has also been a common theme. These studies utilized different methodologies, including meta-analyses, systematic reviews, experimental studies, and quasi-experimental designs, to investigate the relationships between nutrition and specific health conditions.

Nutrition plays a central role in the context of the discussed studies on the healing potential of edible plants and their bioactive natural compounds (5, 6). In this Research Topic, four research papers emphasized the importance of consuming a well-balanced diet rich in nutrients, including vitamins, minerals, antioxidants, and other bioactive compounds found in edible plants. For instance, Salari-Moghaddam et al. identified that the dietary total antioxidant capacity (TAC) could be considered a proper measure for assessing diet quality, highlighting that antioxidants are among the important nutrients in foods included in a high-quality diet. Rawangkan et al. investigated the antimicrobial activity of coffee beans and coffee by-products against drug-resistant *Vibrio cholerae*. The study shows that various phytochemicals, such as caffeic acid and chlorogenic acid, are effective in treating multidrug-resistant *V. cholerae* infections. Barakat et al. demonstrated lentils (*Lens culinaris* M.) as a superfood rich in bioactive phytochemicals that could potentially confer antioxidative, hepatoprotective, and nephroprotective effects. Meanwhile, Saadat et al. consolidated an extensive list of preclinical and clinical evidence on the effects of natural products derived from various plant sources against noxious agents-induced lung injuries, highlighting their therapeutic potential in the clinical management of lung disorders.

There are six publications that investigated the impact of vitamins, namely vitamins C and D, on various health outcomes. These studies focused on their supplementation, dietary intake, and deficiency in relation to various health risks, including colorectal cancer, brain tumor, ischemic stroke, bacterial vaginosis during pregnancy and spinal cord injury recovery. Han et al. conducted a meta-analysis, suggesting that dietary high intake of β-carotene may have a protective effect against colorectal cancer. Zhang W. et al. reviewed the evidence on vitamins and brain tumors, finding that higher intake or serum concentration of vitamins C, β-carotene, and folate may significantly reduce brain tumor risk, providing new perspectives on prevention. Tang et al. reviewed the evidence on vitamin C and ischemic stroke risk, highlighting its protective effects through various mechanisms, including regulating vascular tone and reducing oxidative stress. Ma et al. conducted a meta-analysis, finding that vitamin D deficiency is positively associated with the risk of bacterial vaginosis during pregnancy. Wang et al. reviewed the literature on vitamin D and spinal cord injury (SCI), revealing a high prevalence of vitamin D insufficiency in SCI patients, which may impair functional restoration. Vitamin D supplemental treatment could potentially aid post-injury rehabilitation and has neuroprotective effects.

Lastly, Jittat et al. conducted a quasi-experimental study, finding that oral multi-vitamin multi-mineral (MVMM) supplement formulations, (1) Hydro-Cell-Key (HCK) granule and (2) VTL-7 capsule, increased serum levels of vitamin D and β-carotene. The study suggested that these formulations could be a good reference for future studies on micronutrient supplementation, primarily benefitting those individuals with vitamin A or D deficiency. Collectively, these research papers contribute valuable insights into the potential roles of vitamins in promoting health and preventing diseases. These findings underscore the importance of ensuring adequate vitamin intake for overall wellbeing and highlight potential avenues for therapeutic interventions. However, further research and well-designed clinical trials are necessary to establish definitive causal relationships and optimize vitamin-based interventions for specific health conditions.

The significance of micronutrients cannot be overstated in the realm of nutrition and their impact on overall human health. These essential elements, found in trace amounts within our diets, play a vital role in the proper functioning of our bodies and contribute to overall wellbeing. There were four studies that shed light on the associations between micronutrients and various health outcomes, ranging from thyroid cancer and metabolic syndrome to depression and urologic cancers.

Zhang X. et al. explored the association between iodine nutrition and papillary thyroid cancer (PTC) based on evidence from epidemiological and experimental studies investigating the prevalence, distribution and aggressiveness of PTC in relation to iodine intake. The findings illustrated the U-shaped relationship between iodine and papillary thyroid cancer, indicating the importance of maintaining an optimal and balanced intake of iodine to mitigate potential health risks. Meta-analyses were performed on observational studies to examine the associations between dietary micronutrient intake and metabolic syndromes and depression. Ding et al. unveiled the inverse associations between dietary zinc intake and metabolic syndrome. Similarly, Ding and Zhang demonstrated the negative relationship between dietary copper, selenium, and manganese with depression. These studies provide valuable insights into the potential benefits of these micronutrients in promoting metabolic health and emotional wellbeing.

In contrast, Lu et al. revealed differential roles of micronutrients (copper, iron, and zinc) in influencing the risk for urologic cancers using a two-sample Mendelian randomization study. The study genetically predicted that the increase in serum copper and iron levels was causally associated with an increased risk of renal cell carcinoma (RCC). Meanwhile, an increase in serum zinc level was related to decreased risks of RCC but increased risk of prostate cancer. Evidently, these findings indicate that further exploration and well-designed prospective cohort studies are essential in unraveling the intricate roles of micronutrients in health and disease. By gaining a deeper understanding of the roles and interactions of these trace elements, we can refine our nutritional approaches to support health and wellbeing. Empowering individuals with evidence-based information will enable them to make informed dietary choices, ensuring adequate intake of essential micronutrients. Together, micronutrients hold immense potential to be developed into targeted and personalized nutritional interventions, becoming a cornerstone of public health preventive and therapeutic strategies.

The gut microbiome is a key player in mediating the effects of dietary interventions on various health outcomes (7). The gut microbiome refers to the diverse community of microorganisms, including bacteria, viruses, fungi, and other microbes, that reside in the gastrointestinal tract (8). It plays a critical role in various aspects of health, including digestion, metabolism, immune function, and even mental health (9). Probiotics have been shown to modulate the gut-brain axis, the bidirectional communication system between the gut and the brain (10). In the study by Mahboobi et al., the researchers evaluated the effects of probiotic and magnesium co-supplementation on mood, cognition, intestinal barrier function, and inflammation in individuals with obesity and depressed mood. The randomized, double-blind placebo-control trial concluded that probiotic and magnesium co-supplementation resulted in reduced serum C reactive protein in obese and depressed patients. Similarly, Lee et al. reviewed the use of probiotic therapy in metabolic and neurological diseases, emphasizing the role of the gut microbiome in influencing health outcomes.

Besides that, Kanchanasurakit et al. systematically evaluated the effects of synbiotics, probiotics, and prebiotics on liver enzymes and other clinical parameters in patients with non-alcoholic fatty liver disease (NAFLD). The gut microbiome has been linked to the pathogenesis of NAFLD, and these interventions have the potential to alter the gut microbial community, leading to improvements in liver function. Lastly, Kaewdech et al. explored the effect of fiber supplementation on the prevention of diarrhea in hospitalized patients receiving enteral nutrition. Fiber serves as a prebiotic that nourishes beneficial gut bacteria and confers positive effects on colonocytes. The meta-analysis highlighted that specific fiber types, such as mixed soluble/insoluble fiber and hydrolysed guar gum, are associated with a more evident reduction of diarrhea among hospitalized patients receiving enteral nutrition. Therefore, these studies reveal that gut microbiota modulation-based nutritional interventions are promising avenues for disease prevention and management.

While these research papers contribute valuable insights, there are some potential gaps that future studies could address. First, many of these studies are based on observational data, which can show associations but not causation. Future research should focus more on well-designed randomized controlled trials to establish the therapeutic efficacy of specific dietary components or interventions for various acute and chronic diseases. Moreover, the studies predominantly focus on specific dietary components, but the synergistic effects of a balanced diet on overall health remain to be explored further. Another potential gap is the lack of representation of diverse populations in some of the studies. Nutrition and health outcomes can be influenced by genetic, cultural, and lifestyle factors, which could vary across different populations. Conducting research on diverse populations would provide a more comprehensive understanding of the relationships between nutrition and health (11, 12). Furthermore, the studies primarily focused on the impact of dietary components on disease risk or progression. Exploring the mechanisms by which these components exert their effects at the molecular and cellular levels would enhance our understanding of the underlying biology and facilitate targeted interventions.

In summary, the collection of publications reviewed in this Research Topic sheds light on the intricate relationship between dietary factors and various health conditions. By translating these findings into practice and conducting further research in emerging directions, we can harness the power of nutrition to address global health challenges effectively. While providing valuable insights, these studies also highlight potential gaps in knowledge that warrant further investigation. Future research in this area should aim to establish causal relationships, consider diverse populations, explore mechanisms of action, and emphasize the importance of a balanced diet for overall health and disease prevention. Such endeavors would pave the way for more effective and personalized nutritional interventions to improve public health outcomes.

## Author contributions

L-HL: Conceptualization, Validation, Writing—original draft. B-HG: Conceptualization, Validation, Writing—review and editing. PD: Conceptualization, Validation, Writing—review and editing. SS: Conceptualization, Validation, Writing—review and editing.
